# Body composition and physical activity in Italian university students

**DOI:** 10.1186/1479-5876-12-120

**Published:** 2014-05-09

**Authors:** Luciana Zaccagni, Davide Barbieri, Emanuela Gualdi-Russo

**Affiliations:** 1 Department of Biomedical and Specialty Surgical Sciences, University of Ferrara, Corso Ercole I d’Este 32, 44121 Ferrara, Italy

**Keywords:** Body fat, BMI, WSR, Young adults, Physical activity, University students

## Abstract

**Background:**

Increased sedentary lifestyle and prevalence of overweight/obesity are common in western countries. The purposes of this study were (i) to assess the main health-related anthropometric characteristics in a sample of students in relation to sex, amount of physical activity and sport discipline, and (ii) to investigate the accuracy of the Body Mass Index (BMI) and Waist-to-Stature Ratio (WSR) as indicators of body fat percentage (%F) in young adults.

**Methods:**

734 university students, both sexes, participated in the present research. A self-administered questionnaire acquired socio-demographic information (sex, age) and sport participation (hours/week, sport discipline). Anthropometric measurements and grip strength values were acquired according to standardized procedures. Body composition was assessed by means of the skinfold method.

**Results:**

Most students had normal BMI, WSR and %F. There were significant statistical differences in all anthropometric traits between the two sexes. One-way ANOVAs within sex showed statistically significant differences in biceps skinfold, waist circumference (WC), WSR, body density (BD), %F and fat mass (FM) among different levels of physical activity in males; and in weight, BMI, arm girths and fat free mass (FFM) in females. One-way ANOVAs within sex showed statistically significant differences in arm girths, grip strength and FFM among different sport disciplines in males, and in height, weight, BMI, WC, relaxed arm girth, grip strength, FM and FFM in females. Despite the significant and positive correlation of BMI and WSR with %F both indices had poor sensitivity.

**Conclusions:**

Physical activity plays an important role in body composition parameters: the most active males had the least amount of FM and the most active females had the greatest amount of FFM. BMI and WSR are not accurate indices of adiposity in young adults.

## Background

Body composition assessment is used to monitor performance and training in the athletic community, and to verify the health status of the population in general. The Body Mass Index (BMI) is often used to evaluate the weight status, even if it does not discriminate between different components of the overall body mass by definition (BMI = weight/height^2^). Therefore, the adoption of BMI as a predictor of adiposity and of consequent health risk should be used with caution [1], especially with physically active individuals, who usually have a higher body density and fat free mass (FFM) than the general population [2-4]. Body fat percentage (%F) instead is directly correlated with increased health risk, especially for metabolic and cardiovascular diseases [5-9]. Waist-to-Stature Ratio (WSR) and Waist Circumference (WC) are supposed to have greater discriminatory power compared to BMI [10,11] and are more sensitive than BMI as an early predictor of health-related risks [12]. In particular, WSR is probably the most sensitive anthropometric index for the screening of the metabolic syndrome in Mediterranean populations, compared to both BMI and WC [13].

Low levels of physical activity may place individuals at increased risk of obesity and cardiovascular diseases [14]. On the other side, physical activity has been suggested as a means to reduce and control body fatness. More in general, regular physical activity has proved to effectively reduce diverse health risk factors, especially those related to cardiovascular diseases and the metabolic syndrome [15,16]. In particular, the American College of Sports Medicine recommends that adults engage in at least 150 min∙wk^-1^ of moderate intensity cardiovascular exercise and at least 75 min∙wk^-1^ of vigorous intensity training, in order to maintain a sufficient level of cardio-respiratory fitness. Resistance training is also suggested 2–3 d∙wk^-1^[17]. We can therefore assume that these recommendations amount for a total of more than 4 h∙wk^-1^ of moderate-to-intense physical activity.

The purpose of this study was to assess the main health-related anthropometric characteristics of a group of university students, in order to evaluate their relationship with quantity and type of physical activity according to sex. In particular, FFM, %F, WC, WSR, BMI and grip strength were taken into consideration. Furthermore, the accuracy of BMI and of WSR as predictors of %F was evaluated.

## Methods

### Participants and study design

This was a cross-sectional study carried out on a total of 734 university students, 354 females aged 21.5 ± 2.9 yrs (mean ± standard deviation) and 380 males aged 22.1 ± 3.6 yrs, of the School of Sport Science (Faculty of Medicine, University of Ferrara, Italy) who volunteered for the study. The sample was composed of North Italian students (mainly coming from the regions of Emilia Romagna and Veneto). Body image perception was previously assessed on the same sample [18]. The research protocol was approved by the Ethic Committee for Biomedical Research of the University of Ferrara, and all participants provided written informed consent.

A questionnaire on training and physical activity patterns was administered to participants. The mean weekly amount of physical activity was 6.7 ± 4.2 hrs for males and 4.2 ± 3.8 hrs for females; 28 males (7.4% of the total male sub-sample) and 83 females (23.4% of the total female sub-sample) did not practice any sport activity.

### Anthropometric survey

All measures were taken in the Anthropometry Laboratory at the University of Ferrara, during the tutorials for the students of the course of Anthropometry and Ergonomics in the second year of the School of Sport Science.

Standing (H, cm) and sitting heights (SH, cm) were measured to the nearest 0.1 cm using a wall-mounted stadiometer (Magnimeter, Raven Equipment Limited, UK). Weight (W, kg) was measured to the nearest 0.1 kg using a calibrated electronic scale. BMI was calculated as W/H^2^ (kg/m^2^). Skinfold thicknesses at biceps (B Sk) and triceps (T Sk) were measured to the nearest 0.1 cm using a Lange caliper (Beta Technology Inc.). All girths (Waist Circumference WC, Contracted Arm Girth CAG, Relaxed Arm Girth RAG) were measured to the nearest 0.1 cm using a non-metallic and non-stretchable tape. WSR was calculated as WC/H.

All measurements were taken on the left side of the body, according to standardized procedures [19]. During the anthropometric measurements, all participants were barefoot and clothed appropriately.

Left and right hand grip strength was measured to the nearest 0.5 kg by means of a Takei dynamometer (T.K.K. 5001 grip-A Takei scientific instruments Co., LTD, Japan). The highest value of two trials was recorded, after an adequate period of rest between attempts, for each hand.

### Assessment of body composition

Body density (BD) was calculated using Durnin & Womersley [20] equations with two skinfolds (biceps and triceps), according to sex and age of the student. %F was calculated from BD using Siri equation [21]. Fat Mass (FM, kg) was calculated as (%F*W)/100 and FFM (kg) as W-FM.

### Indices and classifications

According to the World Health Organization [22], underweight was defined as BMI < 18.5 kg/m^2^, normal weight as 18.5 kg/m^2^ ≤ BMI < 25 kg/m^2^, overweight as 25 kg/m^2^ ≤ BMI < 30 kg/m^2^, and obesity as a BMI ≥ 30 kg/m^2^. Because of the small number of students with a BMI ≥ 30 kg/m^2^ (only one female and 15 males), they were included in the overweight group for further elaboration. Even if there is widespread consensus on cut-points for weight status, this is not the case for what concern fatness. According to Gallagher et al. [23], %F ≥ 20% (males) and %F ≥ 33% (females) are the cut-points adopted to define overfatness, corresponding to overweight classification using BMI in a population of young adults.

According to the National Institute for Health and Clinical Excellence guidelines, WC ≥102 cm for men and ≥88 cm for women are prerequisite risk factors for the diagnosis of the metabolic syndrome, as WSR ≥ 0.5 for both males and females [12].

### Statistical analysis

All variables were checked for normality and logarithmically (10-based) transformed where necessary (skinfold at biceps and triceps). Results were expressed as mean ± standard deviation. Comparisons between sexes were carried out using a two-sample Student’s t-test for continuous data and a chi-square (χ^2^) test for categorical data.

Subsequently, both females and males were divided into 3 tertiles, according to their level of weekly physical activity: low (≤3 h∙wk^-1^ for females, ≤5 h∙wk^-1^ for males), medium (3 < h∙wk^-1^ < 6 for females, 5 < h∙wk^-1^ < 8 for males) and high (≥6 h∙wk^-1^ for females, ≥8 h∙wk^-1^ for males). One-way ANOVAs were used to assess the differences in anthropometric variables and grip strength among the 3 groups and *post hoc* comparisons were performed using Tukey test.

Correlation analysis between total weekly hours of physical activity and anthropometric variables was carried out.

In order to assess the anthropometric differences among subjects practicing different activities, one-way ANOVA was performed on sports with at least 10 participants: soccer, body building, basketball, swimming and volleyball in males; gymnastics, other gym activities (O.G.A.), ballet, volleyball, swimming and jogging in females. When a significant F value was obtained, post-hoc comparisons were performed by means of Tukey test.

To determine the accuracy of BMI as a measure of overfatness - and therefore of poor health status - participants were classified into one of four categories: 1) overweight and overfat (True Positive, TP), 2) overweight and normal fat (False Positive, FP), 3) normal weight and overfat (False Negative, FN), and 4) normal weight and normal fat (True Negative, TN). The sensitivity, specificity, and predictive values of BMI were calculated for each group. Sensitivity was calculated as the proportion of overfat individuals who were identified as overweight by BMI (i.e. TP/(TP + FN)). Specificity was calculated as the proportion of normal fat individuals who were identified as normal weight by BMI (i.e. TN/(TN + FP)). Positive Predictive Value (PPV) was calculated as the probability that a participant identified as overweight by BMI was truly overfat: PPV = TP/(TP + FP). Negative Predictive Value (NPV) was calculated as the probability that a participant who was identified as normal weight by BMI was normal fat: NPV = TN/(TN + FN) [24]. Test accuracy increases as the total number of FP and FN decreases.

To test the accuracy of WSR as a measure of overfatness, the same procedure was adopted, substituting the overweight category with excessive WSR.

The statistical significance was set at p < 0.05. All analyses were performed using “Statistica” for Windows, Version 11.0 (StatSoft Italia srl, Padua, Italy).

## Results

There were significant differences among all anthropometric traits between sexes (Table 1). Males were on average heavier, taller, leaner and stronger than females and had wider girths. Females had thicker skinfolds than males, as expected [20,25], therefore they had lower BD and higher %F. 72% of males and 89% of females were normal fat, while 27.3% of males and 10% of females were overfat.

**Table 1:** Anthropometric traits by sex

	**Males**	**Females**
**Trait**	**mean ± SD**	**mean ± SD**
H (cm)	177.6 ± 6.3	163.9 ± 6.0
W (kg)	75.6 ± 10.2	58.7 ± 8.2
BMI (kg/m^2^)	24.0 ± 2.8	21.8 ± 2.6
SH (cm)	92.9 ± 3.5	87.0 ± 3.6
T Sk (mm)	10.8 ± 5.0	16.1 ± 5.5
B Sk (mm)	5.5 ± 3.2	8.7 ± 4.5
WC (cm)	81.7 ± 7.3	70.3 ± 6.5
WSR	0.46 ± 0.04	0.43 ± 0.04
CAG (cm)	32.5 ± 3.1	27.4 ± 2.7
RAG (cm)	29.5 ± 3.0	25.7 ± 2.6
RHG (kg)	50.2 ± 8.0	30.8 ± 5.1
LHG (kg)	48.3 ± 8.1	29.2 ± 5.0
BD (g/cc)	1.059 ± 0.011	1.039 ± 0.011
%F	17.3 ± 4.9	26.6 ± 5.2
FM (kg)	13.3 ± 5.1	16.0 ± 4.9
FFM (kg)	62.4 ± 7.4	42.9 ± 4.9

H = height; W = weight; SH = sitting height; T sk = triceps skinfold; B sk = biceps skinfold; WC = waist circumference; WSR = waist-to-stature ratio; CAG = contracted arm girth; RAG = relaxed arm girth; RHG = right hand grip; LHG = left hand grip; BD = body density; %F = body fat percentage; FM = fat mass; FFM = fat free mass.

Only 4 females (1.2% of the sub-sample) had WC ≥ 88 cm and 7 males (2.0% of the sub-sample) had WC ≥ 102 cm; 5% of females and 13% of males had WSR ≥ 0.5.

BMI mean values were in the normal range according to WHO weight status categories [22]. χ^2^ test proved there was a significant difference (p < 0.001) between sexes in weight status distribution. No male student was underweight, compared to 5.6% of females who fell into this category. Most males (71.7%) and females (80.9%) were normal weight. Males were more overweight (24.2%) and obese (4.2%), than females (13.2% overweight and only 0.3% obese).

ANOVAs within male sub-sample with different levels of physical activity (Table 2) show significant statistical differences in biceps skinfold, WC, WSR, BD, %F and FM, supporting the positive effects of physical activity on health-related anthropometric traits. Tukey post-hoc test shows significant differences only between the low and high level groups.

**Table 2:** Anthropometric traits in male sub-samples by level of physical activity

**Males**	**Low (1st tertile)**	**Medium (2nd tertile)**	**High (3rd tertile)**	
**Trait**	**mean ± SD**	**mean ± SD**	**mean ± SD**	***P-*** **value**
H (cm)	177.2 ± 6.2	177.6 ± 6.4	178.1 ± 6.3	0.557
W (kg)	76.4 ± 11.3	75.7 ± 10.5	74.9 ± 8.8	0.507
BMI (kg/m^2^)	24.3 ± 3.3	24.0 ± 3.0	23.6 ± 2.2	0.151
SH (cm)	92.9 ± 3.4	92.8 ± 3.6	93.0 ± 3.6	0.872
T Sk (mm)	11.6 ± 5.7	10.9 ± 4.2	10.0 ± 4.7	0.054
B Sk (mm)	6.0 ± 3.1^a^	5.5 ± 3.3	5.2 ± 3.2	0.043
WC (cm)	83.1 ± 7.9^a^	81.8 ± 8.2	80.3 ± 5.5	0.009
WSR	0.47 ± 0.05^a^	0.46 ± 0.05	0.45 ± 0.03	0.002
CAG (cm)	32.8 ± 3.1	32.3 ± 3.1	32.4 ± 3.2	0.483
RAG (cm)	29.7 ± 2.9	29.4 ± 3.0	29.3 ± 3.0	0.515
RHG (kg)	49.9 ± 7.6	49.9 ± 7.8	50.6 ± 8.5	0.691
LHG (kg)	48.7 ± 7.8	48.1 ± 7.9	48.1 ± 8.6	0.805
BD (g/cc)	1.057 ± 0.012^a^	1.059 ± 0.010	1.061 ± 0.011	0.009
%F	18.2 ± 5.4^a^	17.5 ± 4.3	16.3 ± 4.8	0.009
FM (kg)	14.2 ± 5.9^a^	13.4 ± 4.7	12.4 ± 4.5	0.022
FFM (kg)	62.5 ± 8.0	62.0 ± 7.5	62.6 ± 6.8	0.835

*Note:* Tukey post-hoc test: ^a^p < 0.05 compared with high.

H = height; W = weight; SH = sitting height; T sk = triceps skinfold; B sk = biceps skinfold; WC = waist circumference; WSR = waist-to-stature ratio; CAG = contracted arm girth; RAG = relaxed arm girth; RHG = right hand grip; LHG = left hand grip; BD = body density; %F = body fat percentage; FM = fat mass; FFM = fat free mass.

ANOVAs within female sub-sample with different levels of physical activity (Table 3) show significant statistical differences in weight, BMI, contracted and relaxed arm girths and FFM, supporting the positive effects of physical activity, particularly on FFM. Tukey post-hoc test shows significant differences between the high level group and the other two.

**Table 3:** Anthropometric traits in female sub-samples by level of physical activity

**Females**	**Low (1st tertile)**	**Medium (2nd tertile)**	**High (3rd tertile)**	
**Trait**	**mean ± SD**	**mean ± SD**	**mean ± SD**	***P-*** **value**
H (cm)	163.5 ± 5.9	163.8 ± 5.6	164.4 ± 6.6	0.521
W (kg)	57.9 ± 8.8	57.6 ± 7.5^b^	60.3 ± 8.0	0.019
BMI (kg/m^2^)	21.6 ± 2.9	21.5 ± 2.4^b^	22.3 ± 2.4	0.035
SH (cm)	86.6 ± 3.5	87.0 ± 3.2	87.4 ± 3.9	0.224
T Sk (mm)	16.5 ± 5.4	15.8 ± 6.1	16.1 ± 5.1	0.497
B Sk (mm)	9.2 ± 4.5	8.4 ± 5.1	8.4 ± 3.8	0.253
WC (cm)	70.3 ± 7.9	69.5 ± 6.1	70.7 ± 5.4	0.403
WSR	0.43 ± 0.05	0.43 ± 0.04	0.43 ± 0.03	0.488
CAG (cm)	26.9 ± 2.7^a^	27.1 ± 2.5^b^	28.0 ± 2.7	0.004
RAG (cm)	25.4 ± 2.6^a^	25.5 ± 2.4	26.1 ± 2.6	0.045
RHG (kg)	30.0 ± 5.2	31.0 ± 4.6	31.5 ± 5.3	0.076
LHG (kg)	28.4 ± 5.0	29.4 ± 4.8	29.8 ± 5.1	0.068
BD (g/cc)	1.038 ± 0.011	1.040 ± 0.012	1.039 ± 0.010	0.337
%F	27.2 ± 5.4	26.1 ± 5.6	26.5 ± 4.8	0.337
FM (kg)	16.1 ± 5.2	15.3 ± 4.9	16.3 ± 4.5	0.315
FFM (kg)	41.9 ± 5.0^a^	42.4 ± 4.6^b^	44.1 ± 4.8	0.001

*Note*: Tukey post-hoc test: ^a^p < 0.05 compared with high; ^b^p < 0.05 compared with high.

H = height; W = weight; SH = sitting height; T sk = triceps skinfold; B sk = biceps skinfold; WC = waist circumference; WSR = waist-to-stature ratio; CAG = contracted arm girth; RAG = relaxed arm girth; RHG = right hand grip; LHG = left hand grip; BD = body density;%F = body fat percentage; FM = fat mass; FFM = fat free mass.

Statistical correlations between hours of physical activity and BMI, triceps and biceps skinfolds, WC, WSR, BD, %F and FM were significant (p < 0.05) in males, and biceps skinfold, left and right had grip strength, BD, %F and FFM in females (Table 4).

**Table 4:** Correlation coefficients between anthropometric traits and hours of physical activity in males and females

**Trait**	**Males**	**Females**
H (cm)	0.045	0.038
W (kg)	-0.076	0.042
BMI (kg/m^2^)	-0.114*	0.022
SH (cm)	-0.015	0.048
T Sk (mm)	-0.180**	-0.072
B Sk (mm)	-0.197***	-0.0173**
WC (cm)	-0.163**	-0.021
WSR	-0.170**	-0.048
CAG (cm)	-0.074	0.091
RAG (cm)	-0.073	0.034
RHG (kg)	0.028	0.123*
LHG (kg)	0.003	0.127*
BD (g/cc)	0.212***	0.120*
%F	-0.212***	-0.121*
FM (kg)	-0.200***	-0.063
FFM (kg)	0.033	0.131*

***p < 0.001 **p < 0.01 *p < 0.05.

H = height; W = weight; SH = sitting height; T sk = triceps skinfold; B sk = biceps skinfold; WC = waist circumference; WSR = waist-to-stature ratio; CAG = contracted arm girth; RAG = relaxed arm girth; RHG = right hand grip; LHG = left hand grip; BD = body density; %F = body fat percentage; FM = fat mass; FFM = fat free mass.

ANOVAs between sport disciplines with more than 10 participants in males (Table 5) show significant statistical differences in relaxed and contracted arm girths, left and right hand grip strength and FFM. Tukey post-hoc test shows significant differences between body building and other sports, especially soccer, for all the traits above.

**Table 5:** Anthropometric traits by sport in males

**Males**	**Soccer N = 132**	**Swimming N = 25**	**Basketball N = 26**	**Bodybuilding N = 41**	**Volleyball N = 13**	
**Traits**	**mean ± SD**	**mean ± SD**	**mean ± SD**	**mean ± SD**	**mean ± SD**	**p**
H (cm)	177.0 ± 6.1	177.8 ± 6.0	180.0 ± 6.9	176.9 ± 6.9	178.2 ± 6.3	0.298
W (kg)	74.5 ± 9.3	74.5 ± 9.0	75.7 ± 9.9	77.7 ± 9.3	78.4 ± 13.5	0.295
BMI (kg/m^2^)	23.8 ± 2.4	23.6 ± 2.6	23.5 ± 3.2	24.8 ± 2.4	24.8 ± 4.7	0.141
SH (cm)	92.8 ± 3.6	93.4 ± 3.0	94.0 ± 3.6	92.5 ± 3.7	92.0 ± 3.3	0.391
T Sk (mm)	10.8 ± 5.1	11.3 ± 5.5	10.9 ± 3.9	9.5 ± 4.2	11.0 ± 4.0	0.452
B Sk (mm)	5.9 ± 3.8	6.0 ± 3.3	4.9 ± 2.1	4.6 ± 2.0	4.9 ± 1.9	0.145
WC (cm)	81.3 ± 6.3	81.0 ± 5.8	82.9 ± 6.6	81.9 ± 6.8	83.3 ± 14.0	0.184
WSR	0.46 ± 0.04	0.46 ± 0.03	0.46 ± 0.04	0.46 ± 0.04	0.47 ± 0.09	0.763
CAG (cm)	31.5 ± 2.7	33.0 ± 2.5	31.9 ± 2.5	36.0 ± 3.2^a^	32.1 ± 2.8	0.000
RAG (cm)	28.5 ± 2.6	29.7 ± 2.4	28.8 ± 2.5	32.4 ± 3.1^a^	29.4 ± 2.4	0.000
RHG (kg)	47.9 ± 7.7	50.2 ± 5.6	50.2 ± 6.8	55.0 ± 8.5^b^	48.9 ± 7.1	0.000
LHG (kg)	45.6 ± 7.4	48.4 ± 4.1	49.9 ± 6.6	52.5 ± 9.5	49.5 ± 8.4	0.000
BD (g/cc)	1.059 ± 0.011	1.058 ± 0.011	1.060 ± 0.008	1.062 ± 0.011	1.059 ± 0.011	0.618
%F	17.4 ± 4.9	18.0 ± 4.8	17.1 ± 3.7	16.2 ± 4.8	17.6 ± 4.8	0.620
FM (kg)	13.1 ± 5.0	13.7 ± 4.9	13.0 ± 3.6	12.7 ± 4.4	14.2 ± 5.6	0.837
FFM (kg)	61.2 ± 6.6	60.9 ± 6.1	62.4 ± 8.4	65.2 ± 8.2^b^	64.2 ± 10.2	0.035

Tukey post-hoc test: ^a^Bodybuilding vs all other sports p < 0.001 ^b^Bodybuilding vs soccer p < 0.05.

H = height; W = weight; SH = sitting height; T sk = triceps skinfold; B sk = biceps skinfold; WC = waist circumference; WSR = waist-to-stature ratio; CAG = contracted arm girth; RAG = relaxed arm girth; RHG = right hand grip; LHG = left hand grip; BD = body density; %F = body fat percentage; FM = fat mass; FFM = fat free mass.

Body builders had the highest BMI – similar to that of volleyball players -, arm girths, right and left hand grip strength, BD and FFM, and the lowest H, skinfold thicknesses, %F and FM. Soccer players had the lowest W, arm girths and hand grip. Volleyball players had the highest W, WC and FM, and the lowest SH. Basketball players had the highest H and SH, and the lowest BMI. Swimmers had the thickest skinfolds and the highest %F, the lowest WC, BD, and FFM.

ANOVAs between sport discipline with more than 10 participants in females (Table 6) show significant statistical differences in height, weight, BMI, WC, RAG, left and right hand grip strength, FM and FFM. Tukey post-hoc test shows significant differences between volleyball players, gymnasts and dancers for the traits above. Volleyball players had the highest H, SH, W, BMI, triceps skinfold, girths, hand grip strength, %F and FFM. Gymnasts were the shortest and lightest and had the greatest BD, the lowest SH, skinfold thickness, WC, %F, FM and FFM. Dancers had the smallest arm girths (RAG values being similar to those of gymnasts) and grip strength.

**Table 6:** Anthropometric traits by sport in females

**Females**	**Gymnastics N = 19**	**O.G.A. N = 50**	**Swimming N = 39**	**Jogging N = 16**	**Ballet N = 47**	**Volleyball N = 47**	**p**
**Traits**	**mean ± SD**	**mean ± SD**	**mean ± SD**	**mean ± SD**	**mean ± SD**	**mean ± SD**	
H (cm)	161.2 ± 6.0	163.0 ± 5.8	164.7 ± 6.6	163.7 ± 4.7	163.4 ± 4.5	166.2 ± 7.0^a^	0.033
W (kg)	54.9 ± 5.9	58.3 ± 8.7	58.0 ± 8.1	58.3 ± 9.4	57.1 ± 6.1	62.8 ± 7.9^a,b^	0.002
BMI (kg/m^2^)	21.0 ± 2.0	21.9 ± 2.9	21.3 ± 2.3	21.7 ± 2.7	21.4 ± 1.9	22.7 ± 2.3	0.049
SH (cm)	86.3 ± 3.0	86.4 ± 3.7	87.1 ± 4.0	87.3 ± 3.1	87.1 ± 3.3	88.4 ± 4.0	1.635
T Sk (mm)	14.3 ± 4.1	14.9 ± 5.8	16.0 ± 6.5	15.2 ± 4.1	15.8 ± 4.7	17.5 ± 5.0	0.177
B Sk (mm)	6.6 ± 2.4	8.9 ± 4.6	9.7 ± 5.0	9.6 ± 3.6	7.6 ± 3.5	8.7 ± 3.5	0.083
WC (cm)	67.1 ± 4.8	69.4 ± 6.1	70.6 ± 7.6	70.6 ± 7.1	68.3 ± 4.1	71.9 ± 5.0^a^	0.023
WSR	0.42 ± 0.03	0.43 ± 0.04	0.43 ± 0.05	0.43 ± 0.04	0.42 ± 0.02	0.43 ± 0.03	0.359
CAG (cm)	26.9 ± 1.2	27.4 ± 2.7	27.2 ± 2.6	27.1 ± 2.5	26.6 ± 2.4	28.3 ± 2.3	0.063
RAG (cm)	24.9 ± 1.5	25.5 ± 2.6	25.7 ± 2.7	25.5 ± 2.8	24.9 ± 2.2	26.6 ± 2.3^b^	0.033
RHG (kg)	30.1 ± 4.6	31.8 ± 5.4	30.7 ± 4.8	31.3 ± 4.3	28.3 ± 3.6^c^	31.8 ± 5.3^b^	0.008
LHG (kg)	29.3 ± 4.8	29.8 ± 5.5	28.7 ± 4.6	29.9 ± 5.1	26.7 ± 3.9^c^	30.1 ± 4.8^b^	0.018
BD (g/cc)	1.043 ± 0.009	1.040 ± 0.010	1.038 ± 0.014	1.038 ± 0.009	1.040 ± 0.010	1.037 ± 0.010	0.333
%F	24.4 ± 4.0	26.1 ± 5.4	26.8 ± 6.3	26.9 ± 4.2	26.0 ± 4.5	27.6 ± 4.5	0.329
FM (kg)	13.2 ± 2.1	15.6 ± 5.1	15.8 ± 5.3	16.0 ± 4.4	14.9 ± 3.9	17.8 ± 4.4^a^	0.009
FFM (kg)	40.9 ± 4.6	43.1 ± 5.2	42.5 ± 5.2	42.2 ± 5.9	41.6 ± 3.1	45.4 ± 4.4^a,b^	0.003

O.G.A. = other gym activities.

Tukey post-hoc test: ^a^volleyball versus gymnastics p < 0.05 ^b^volleyball vs ballet p < 0.01 ^c^ballet vs other gym activities p < 0.05.

H = height; W = weight; SH = sitting height; T sk = triceps skinfold; B sk = biceps skinfold; WC = waist circumference; WSR = waist-to-stature ratio; CAG = contracted arm girth; RAG = relaxed arm girth; RHG = right hand grip; LHG = left hand grip; BD = body density; %F = body fat percentage; FM = fat mass; FFM = fat free mass.

**Figure 1:**
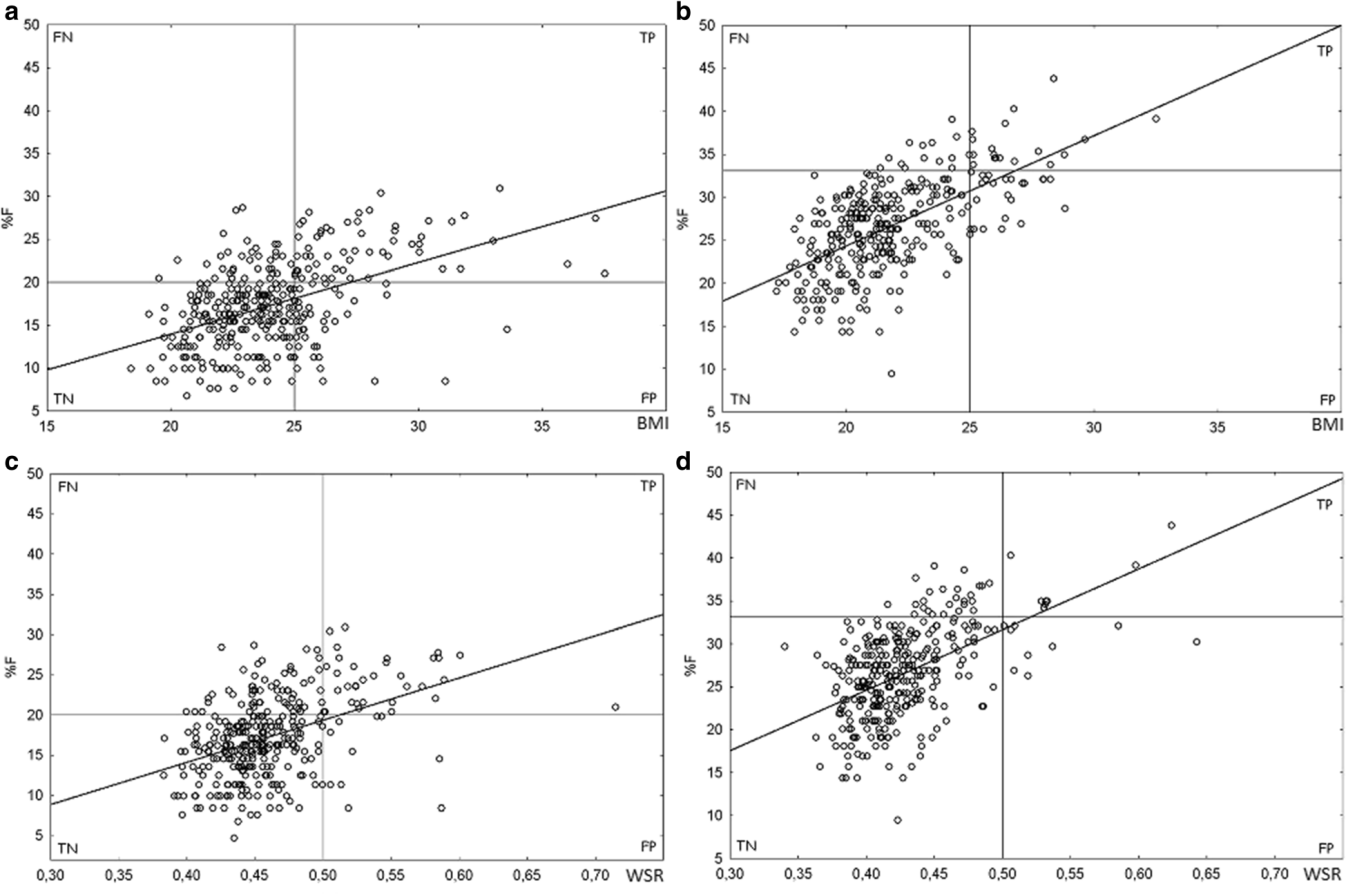
Scatterplot of anthropometric indices BMI ([a] and [b]) and WSR ([c] and [d]) and %F for each male ([a] and [c]) and female ([b] and [d]) study participant - in each scatterplot the four quadrants are labelled FN (false negative), TP (true positive), TN (true negative), and FP (false positive) to illustrate the correct classifications and misclassifications.

A significant positive correlation between BMI and %F was found in males (r = 0.476, p < 0.001), but it did not reach significance in basketball players (p = 0.300) and body builders (p = 0.906). In fact, one third of the subjects who were classified as overweight according to BMI, but who were actually normal fat, practiced body building. Twelve percent of total participants fell within the FP quadrant and 10% in the FN one (Figure 1[a]). Sensitivity was 0.62 and specificity was 0.83, while PPV was 0.58 and NPV was 0.85. A significant positive correlation between BMI and %F was found in females (r = 0.622, p < 0.001) but it did not reach significance in gymnasts (p = 0.752). Seven percent were classified as FP and 4% as FN (Figure 1[b]). Sensitivity was 0.59 and specificity was 0.92, while PPV was 0.45 and NPV was 0.95. Therefore, sensitivity was poor for both sexes, reflecting the fact that the individuals who were at the same time classified as overfat (according to their %F) and overweight (according to their BMI) were only a small proportion of those who were actually overfat. Also PPV of BMI was poor, because really fat individuals were about a half of those who were classified as overweight.

A significant positive correlation between WSR and %F was found in males (r = 0.439, p < 0.001). Four percent of total participants fell in the FP quadrant and 17% in the FN one (Figure 1[c]). Sensitivity was 0.36 and specificity was 0.95, while PPV was 0.73 and NPV was 0.80. A significant positive correlation between WSR and %F was found in females (r = 0.527, p < 0.001). Three percent of total participants fell within the FP quadrant and 8% in the FN one (Figure 1[d]). Sensitivity was 0.24 and specificity was 0.97, while PPV was 0.47 and NPV was 0.92. Therefore, sensitivity was poor for both sexes, and PPV was poor especially in females.

## Discussion

In the present study, we found a different trend in the two sexes in relation to training volume: female students performing more hours of weekly physical activity had a significantly higher amount of FFM compared to the less active individuals, while male students showed a lower %F and FM. A study by Westerterp et al. [26] found a negative correlation between energy expenditure and %F in males, but not in females. Also, a negative correlation between physical activity and FM was found in males, but not in females [27]. Even if FM can be reduced by means of increased physical activity, females seem to compensate for excess energy expenditure with added energy intake. Since women tend to preserve their energy balance more than men [28], FM loss can be not significant. Increased caloric intake could also justify added FFM, as in the present research.

The different behaviours may be consistent with both sex-related differences and sport preferences. The examined females are more often than males engaged in individual sports and in disciplines with a relevant aesthetic component (gym activities, ballet, gymnastics). Males are more often than females engaged in team sports (soccer, basketball, volleyball) or in strength-related activities, like body building, both involving intense repeated efforts, which have been positively correlated to fat loss [29,30]. So the different adaptation could be sport-related. Body building determines an evident increase on muscle hypertrophy, which is significant in comparison to soccer. This fact may contribute to the limited accuracy of BMI as an index of body fatness and general health status, since body builders have a high BMI, close to overweight, even if they have the lowest %F in our sample. Also, females are less physically active, on average, therefore it can be hypothesized that physical adaptation in response to moderate physical activity can be correlated to increased muscle mass, and, vice-versa, that physical adaptation in response to high volume of weekly physical activity can be correlated to reduced %F.

The variance in weekly hours of physical activity within the sample determined significant differences in body composition, and showed the limits of BMI and WSR as indices of adiposity. Intersecting BMI values with %F, we have obtained important indications on its limited applicability in a sample of young adults with different levels of weekly training hours.

The analysis of specificity and sensitivity showed that neither BMI nor WSR can be considered accurate indices of the health status of the population of young adults because they are not consistent measures of body fatness. In fact, both BMI and WSR had good specificity versus %F, but low sensitivity, suggesting that a significant percentage of overfat individuals were classified as normal according to BMI or WSR.

A possible limiting factor of the present study is that physical activity assessment (weekly training hours and type of sport) was based only on a self-reported questionnaire. Also, the training volume does not account for training intensity and quality (mainly aerobic, anaerobic etc.). A lower volume of weekly training hours involving a strenuous practice may have more significant outcomes than a higher volume with a less intense effort, in particular for what concern body composition. Moreover, it must be highlighted that the skinfold-thickness technique is an indirect method for assessing body composition, based on population-specific predictive equations. Although relatively inexpensive, non-invasive, and widely used in sportsmen, its accuracy cannot be granted especially in individuals with adipose tissue that is not well separated from the underlying muscle [31].

## Conclusions

This study examined a large sample of Italian university students from the same geographical area by means of rigorous anthropometric procedures.

In conclusion, a different behaviour was highlighted in the two sexes in relation to weekly amount of physical activity: males mainly showed a decrease in %F, whereas females showed an increase in FFM, which could be explained by a stronger tendency to maintain energetic balance or by different sport preferences.

BMI and WSR have been suggested as indirect measures of %F, because of the ease with which they can be collected. The present study confirms their low accuracy. In fact, in females, misclassification (FP + FN) was 11% for both BMI and WSR. In males, misclassification was 22% for BMI and 21% for WSR. Therefore, regardless of the fact that WSR has been proposed as a better index of adiposity than BMI, both indices show similar low accuracy and they cannot be considered reliable predictors of body fatness, especially in young males. Greater accuracy can be found in females, possibly because of lower overall FFM compared to males. In fact, high FFM contributes to increased BMI, without any real detrimental effect (e.g. in body builders).

The present study confirms that an active lifestyle, including regular weekly physical activity, is significantly correlated to body composition parameters.

## Competing interests

The authors declare that they have no competing interests.

## Authors’ contributions

EG-R and LZ have planned the study, ZL and BD participated to the data collection and did all the statistical analysis. All authors were involved with data interpretation, critical revisions of the paper. All authors read and approved the final manuscript.
